# EDITORIAL COMENT: RETROPERITONEOSCOPIC PYELOLITHOTOMY: A GOOD ALTERNATIVE TREATMENT FOR RENAL PELVIC CALCULI IN CHILDREN

**DOI:** 10.1590/S1677-5538.IBJU.2015.0242.1

**Published:** 2016

**Authors:** Lucas Wiegand

**Affiliations:** 1 University of South Florida Morsani College of Medicine, Tampa, FL, USA

This video Cezarino et al. (1) was very well about a retroperitoneal laparoscopic pyelolithotomy. While executed flawlessly, the applicability of this video to the average urologic practice is limited because of the other treatments readily available that are usually exhausted prior to laparoscopic stone surgery. Generally, open and robotic/laparoscopic approaches are used in combination with procedures for coexisting anatomic abnormalities. PCNL, ESWL, and ureteroscopy are safe, available at most institutions and should be utilized in the pediatric population as first-line therapy (2). Despite this, the video does add to the literature and will be needed as a guide for those rare cases when laparoscopic stone surgery is needed.
